# Inhibiting TRF1 upstream signaling pathways to target telomeres in cancer cells

**DOI:** 10.15252/emmm.201910845

**Published:** 2019-06-13

**Authors:** Julien Cherfils‐Vicini, Eric Gilson

**Affiliations:** ^1^ Université Côte d'Azur (UCA) Centre National de la Recherche Scientifique (CNRS) UMR7284 Institut National de la Santé et de la Recherche Médicale (INSERM) U1081 Institute for Research on Cancer and Aging Nice (IRCAN) Nice France; ^2^ Department of Medical Genetics Archet 2 Hospital CHU of Nice Nice France

**Keywords:** Cancer, Pharmacology & Drug Discovery

## Abstract

In the context of tumorigenesis, telomere shortening is associated with apparent antagonistic outcomes: On one side, it favors cancer initiation through mechanisms involving genome instability, while on the other side, it prevents cancer progression, due to the activation of the DNA damage response (DDR) checkpoint behaving as a cell‐intrinsic proliferation barrier. Consequently, telomerase, which can compensate for replicative erosion by adding telomeric DNA repeats at the chromosomal DNA extremities, is crucial for cancer progression and is upregulated in nearly 90% of human cancers. Therefore, telomeres are considered potential anti‐cancer targets and, to date, most of the studies have focused on telomerase inhibition. However, the development of clinically efficient telomerase targeting therapies is still in its infancy. In this context, the findings reported in this issue of *EMBO Molecular Medicine* by Bejarano *et al* (2019) open new avenues for alternative telomere therapies.

In human cells, telomeric chromatin is organized into a terminal loop (t‐loop), nucleosomes, the non‐coding RNA TERRA, the protein complex shelterin, and a network of nuclear factors. The shelterin complex is essential for telomere protection and comprises six subunits: Three subunits bind telomeric DNA (TRF1, TRF2, and POT1), while the three others establish protein–protein contacts: RAP1 with TRF2, TIN2 with TRF1, TRF2, and TPP1 with TIN2 and POT1. Each shelterin subunit has a specific role in telomere protection, i.e., TRF1 prevents replication stress, TRF2 blocks ataxia telangiectasia‐mutated (ATM) signaling and non‐homologous end joining (NHEJ), while POT1 blocks ataxia telangiectasia and Rad3‐related (ATR) signaling.

A wealth of recent findings points toward shelterin as a valuable alternative to telomerase to fight cancer. POT1 mutations, as well as increased dosages of TRF1 and TRF2, are observed in several types of human malignancies (Nakanishi *et al*, 2003; Ramsay *et al*, 2013; Bejarano *et al*, 2017; Cherfils Vicini *et al*, 2019). Consistently, the TRF1 and TRF2 expression is regulated by key cancer signaling pathways such as canonical Wnt (Diala *et al*, 2013), WT1 (El Maï *et al*, 2014), and PI3K/AKT (Bejarano *et al*, 2017). An overexpression of TRF1 and TRF2 has been implicated in various pro‐tumorigenic mechanisms including initiation and progression, migration, metastasis, angiogenesis (El Maï *et al*, 2014; Picco *et al*, 2016; Zizza *et al*, 2019), cancer stemness (Bejarano *et al*, 2017), telomere maintenance (García‐Beccaria *et al*, 2015; Bejarano *et al*, 2017), or immunosurveillance bypass (Biroccio *et al*, 2013; Cherfils Vicini *et al*, 2019), making these shelterin subunits interesting multi‐hit targets for cancer treatment.

In this issue of *EMBO Molecular Medicine*, Bejarano *et al* (2019) identified small compounds targeting TRF1 using an FDA‐approved library to screen for TRF1 expression and localization. Several of the drugs downregulating TRF1 expression interfere with common cancer signaling pathways including ERK and MEK, Aurora, CDK, PLK1, HSP90, mTOR, RTK, or chemotherapy drugs like gemcitabine and docetaxel. Treatment of lung cancer and glioblastoma cells with these compounds triggered DDR activation at telomeres and telomere replication defects. In patient‐derived glioblastoma stem cells (GSC), these TRF1 inhibitors reduced stemness *in vitro*. Importantly, the authors showed that TRF1 is a direct substrate for ERK2 and bRAF, two kinases of the Ras pathway, and mTOR. Among the 13 residues they identified for TRF1 phosphorylation by ERK2 and the 4 for bRAF, T330 appears critical for both bRAF and ERK2 phosphorylation as well as for TRF1‐telomere complex formation. Inhibiting T330 phosphorylation is sufficient to increase telomere uncapping, to reduce proliferation and stemness *in vitro*, and to reduce tumorigenesis *in vivo*. Finally, combined treatment with PI3K inhibitors and ERK2 or bRAF inhibitors showed synergistic effects *in vitro* and *in vivo* on patient‐derived glioblastoma mouse model, showing that TRF1 targeting via phosphorylation inhibition could be a promising strategy.

Interestingly, data from Bejarano *et al* (2019) make a direct connection between TRF1 phosphorylation by common cancer signaling pathways, telomere protection, and cancer treatment. This link is certainly not limited to TRF1, since TRF2 can also be phosphorylated by the ERK1/2 kinases and interacts with Ras signaling to bypass DDR in cancer cells (Biroccio *et al*, 2013; Picco *et al*, 2016). This is in agreement with the partial rescue of telomere protection reported in Bejarano *et al*, through TRF1 overexpression in cells treated with ERK inhibitor, which suggests that other telomere components are involved. In this context, testing known molecules inhibiting Ras farnesylation could be an interesting anti‐telomere strategy.

Taken together, these findings indicate that known drugs targeting common cancer signaling pathways can act through shelterin downregulation, suggesting valuable drug combinations in future chemotherapies and precise medicine against cancer (Fig 1).

**Figure 1:**
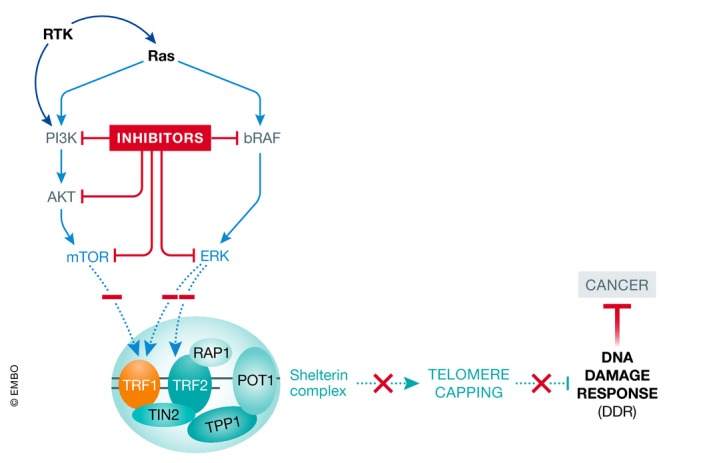
Telomere uncapping and anti‐cancer effect induced by Ras signaling inhibition The inhibition of FDA‐approved inhibitors targeting key molecules downstream of Ras inhibits the phosphorylation of the shelterin TRF1 and TRF2. This inhibition of phosphorylation triggers telomere uncapping, DDR activation, and anti‐cancer effect *in vitro* and *in vivo*.
